# Clathrate Hydrates of Greenhouse Gases in the Presence of Natural Amino Acids: Storage, Transportation and Separation Applications

**DOI:** 10.1038/s41598-018-26916-1

**Published:** 2018-06-04

**Authors:** Pinnelli S. R. Prasad, Burla Sai Kiran

**Affiliations:** Gas Hydrate Division, CSIR–National Geophysical Research Institute (CSIR–NGRI), Hyderabad, 500 007 India

## Abstract

Storage of greenhouse gases in the form of gas hydrates is attractive and is being pursued rigorously in recent times. However, slow formation rate and inefficient water to hydrate conversion are the main hindering factors. In this report, we examine the role of two amino acids (0.5 wt%), l-methionine (l-met) and l-phenylalanine (l-phe) on the formation of gas hydrates using methane (CH_4_), carbon dioxide (CO_2_) and their mixtures as guest molecules. Experiments are conducted under non-stirred and isochoric configurations. The hydrate conversion efficiency of both amino acids is identical for hydrates formed with CH_4_ and mixture of (CO_2_+CH_4_). However, the hydrate conversion is significantly less in CO_2_ hydrates in l-phe system. Addition of amino acids to the water dramatically improved the kinetics of hydrate formation and 90% of maximum gas uptake in hydrate phase occurred in less than an hour. The water to hydrate conversion is also very efficient (>85%) in the presence of amino acids. Therefore, the amino acids containing systems are suitable for storing both CH_4_ and CO_2_ gases. The gas hydrates were characterised using powder x-ray diffraction (XRD) and Raman spectroscopic measurements. These measurements indicate the formation of sI hydrates and encasing of gas molecules as guests.

## Introduction

Natural gas hydrates, crystalline ice-like solids, often, found in certain oceanic sediments and permafrost regions across the globe. The ensemble of liquid water and some gaseous molecules transform into solid gas hydrates occur in some parts, where moderately high pressure and low-temperature conditions co-exist. With as much as 163 units of gas contained per unit volume of gas hydrate, these concentrated fuel gas-solids are reviewed as new-energy resources. Although the current estimate of the amount of gas contained in this form is promising (nearly double to all other carbon sources), the process development for safer and sustainable extraction is challenging, mainly because of adverse p-T conditions. Nevertheless, even 10% of gas extraction from this source will be sufficient for global energy requirements for next 100 years or so at the current level of consumption^1–3^.

Certain unique properties, like large gas-storage capacity and latent heat associated with the gas hydrate transformation, has opened up several exciting applications. The hydrate based technology is helpful in gas storage purposes for natural gas, methane, hydrogen and carbon dioxide^4–6^. Required pressure-temperature conditions for hydrate stability are modest in contrast to other forms of gas storage methods, namely gas compression and liquefaction of natural gas. The gas hydrates, particularly with methane and carbon dioxide, are abnormally stable outside its thermodynamic boundary and this property is useful in gas transportation. Recently, some researchers demonstrated the use of mixed hydrates for methane gas storage and transportation applications^7,8^. The heat release during hydrate formation/dissociation is explored in cool-energy storage in air-conditioning applications^9^.

The research on gas hydrates initially started purely as a scientific curiosity, but subsequently, it was identified as one of the leading cause for blockages in petroleum/gas carrying pipeline network. Later on, research efforts were intensified to find a solution for the purpose. Traditionally three types of inhibiting materials namely thermodynamic (THI), kinetic (KHI) and anti-agglomerates are being tested to control the formation of such blockages. Use of THIs shifts the phase boundary curve of hydrate system to the left, thereby driving the formation to lower temperatures and high-pressure conditions. Traditional THIs like alcohols and glycols permanently inhibit the formation of hydrates but this approach requires the injection of vast amounts of THIs, and additional facilities to deliver and recover them adds to the process cost. The KHIs (water-soluble polymers), on the other hand, considerably delays the hydrate formation in the time domain. The third class of materials anti-agglomerates prevents the agglomeration of hydrate particulates, and with small sized hydrates, the flow related issues are minimised. These materials are relatively new to gas hydrates systems, and some proteins and bio-extracts from living organisms are being examined. The need for inhibitors originates in preventing the hydrate growth, however for some well-known applications such as gas storage and transportation one should think of promoting the hydrates. Similar materials upon using in low-dosages can serve as promoters. Therefore the research on different materials even though they do not directly take part in actual hydrate cage formation is being pursued rigorously^10–13^.

Recently, aqueous solutions of amino acids (AAs) have been demonstrated as superior thermodynamic inhibitors for both methane and carbon dioxide hydrates^14–26^. On the other hand, they can also significantly promote the hydrate growth under some conditions, particularly in lower concentrations. The amino acids are attractive because of their ability to mix with water through hydrogen bonding, non-toxic and bio-friendly nature. There are twenty different essential amino acids in nature and are found in proteins. By the propensity of the side chain to interact with polar solvents like water, they are classified as hydrophobic, polar or charged^27^. Amino acids are zwitterions having both amino and carboxylic groups, and their isoelectric point (pI) critically depends on the side chain^27^.

Sa *et al*.^14–18^ have conducted extensive studies of gas hydrates with CO_2_, CH_4_ and natural gas (NG) using different amino acid solutions in the concentration range of 0.01–3.0 mol%. These authors used different amino acids such as glycine, alanine, proline, valine, leucine, isoleucine, phenylalanine, histidine, and serine etc., for their study. The essential observations from those studies are: Proline and valine were found to show the highest inhibition impact for methane and carbon dioxide hydrates, respectively. The history of water, concentration of the additive, length and nature of side chain of the amino acid can have a noticeable influence on hydrate inhibition property. The amino acids kinetically reduce carbon dioxide consumption compared to pure water via local water perturbation. Roosta *et al*.^19^ also investigated the effect of amino acids (0.5 to 2 wt%) on the hydrate formation in CO_2_ – H_2_O system. Those experiments revealed that the hydrate growth rate decrease in the following order histidine > glycine > proline~serine~threonine > glutamine. Further, inhibition also is more at higher concentrations. Bavoh *et al*.^20^, have also studied the influence of amino acids (5 to 20 wt%) on CO_2_ hydrates. They found THI effect in the following order glycine > alanine > proline > serine > arginine. The highest measured temperature depression is 1.83 K for glycine (10 wt%). They also reported an increase in the inhibition effect by increasing the concentration of glycine. Bavoh *et al*.^21^ have also examined the effect of valine and arginine (1 to 5 wt%) on methane hydrates. They found a slightly lower hydrate dissociation temperature (0.5 K) in the presence amino acids, but with enhanced gas uptake rate. At least ten times higher methane gas uptake was noted in the valine bearing system. Interestingly most of these were conducted in stirred reactors. In another study Cai *et al*.^22^, reported some amino acids, such as l-methionine, l-norvaline and l-norleucine have a large CO_2_ gas storage potential in the form of hydrates and with faster gas uptake, even under non-stirred configuration. We also examined hydrate formation in CO_2_ – H_2_O system, under similar experimental conditions, using some amino acids such as l-methionine, l-cystine and l-valine and found that the gas storage is high, whereas, it is less for l-phenylalanine and l-theornine containing systems^23^. Further, Liu *et al*.^24^, found that the leucines, particularly under lower concentrations (0.1 to 1 wt%), are effective promoters for methane gas in hydrated form with a high formation rate and a high capacity. These authors have also reported that l-methionine, l-tryptophan, l-phenylalanine, l-arginine, l-glutamic acid and l-histidine can promote the formation process of methane hydrate, however, to some lesser extent. Later on, some other researchers have also used some of these amino acids to examine their influence on methane hydrates and have also examined morphology of the hydrates^25,26^.

In this study, we have examined the hydrate formation ability in two amino acid solutions using CO_2_, CH_4_ and their mixtures. We particularly chose l-methionine (l-met) and l-phenylalanine (l-phe) because of their distinct variations in the side chains, i.e., these have aliphatic and aromatic groups in the side chains, but have been classified as hydrophobic^27^. Also, they are the essential amino acids, which human body cannot produce, and therefore have to be maintained through nutrition and diet. We observed that the aqueous solution of 0.5 wt% l-met is a good kinetic promoter for gas hydrates with CO_2_ and CH_4_ as guest molecules. The aqueous solution of 0.5 wt% l-phe, on the other hand, is an efficient kinetic promoter for CH_4_ hydrates, while it is inefficient for CO_2_ hydrates. Such contrasting behaviour is explored for hydrate formation with mixed gas. Structural stability of hydrates, particularly, in the metastable region is examined for plausible gas storage and transportation applications.

## Results and Discussion

### Hydrate synthesis

**Figure 1:**
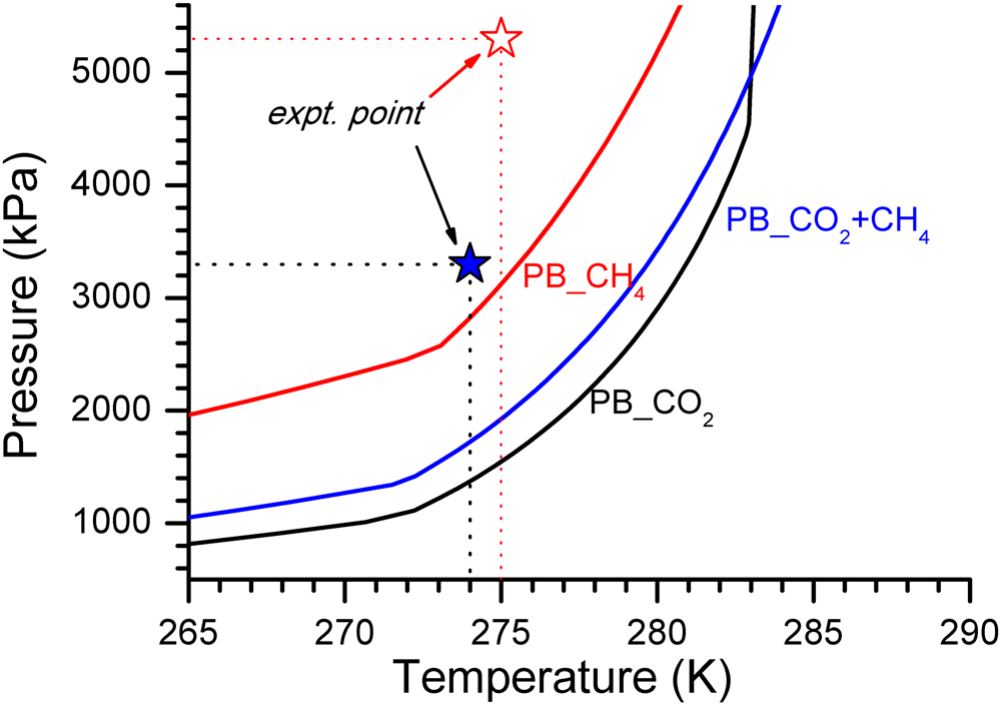
Schematic diagram depicting the experimental conditions on the pressure – temperature (p-T) trajectory for hydrate forming systems. Phase boundary curve for (54% CO_2_ + 46% CH_4_) gas mixture is shown.

The gas hydrates were synthesised in a standard non-stirred configuration in constant volume mode. The process was investigated at different initial gas pressures in view of the differences in the phase boundary conditions. Figure 1 shows a schematic view of the experimental conditions with CO_2_, CH_4_ and their mixtures i.e., (54% CO_2_ + 46% CH_4_), As shown in the figure, initial pressure for CO_2_ and CO_2_+CH_4_ (mixture), is lower to ensure them to be in the gas phase. The operational pressure for CH_4_ hydrates is chosen at ~5300 kPa to ensure similar driving force in all the hydrate-forming systems. Table 1, summarizes the feed-gas composition and observed gas uptake during hydrate conversion process.

**Table 1:** Table showing the feed gas composition, gas consumption in hydrate phase and the conversion of water to hydrate in the presence of 0.5 wt% l-met and l-phe.

Composition of feed gas		Gas uptake (mol/mol)		Conversion (%)^a^	
X_CO2_	X_CH4_	l-met	l-phe	l-met	l-phe
0	100^b^	0.137 (±0.004)	0.147 (±0.007)	86.28	92.25
0	100	0.030 (±0.004)	0.050 (±0.01)	18.87	31.45
12	88	0.070 (±0.01)	0.080 (±0.009)	44.03	50.32
22	78	0.090 (±0.01)	0.080 (±0.012)	57.32	50.96
33	67	0.100 (±0.004)	0.110 (±0.03)	64.10	70.51
54	46	0.120 (±0.004)	0.131 (±0.006)	77.96	84.73
65	35	0.120 (±0.004)	0.113 (±0.005)	78.43	73.85
80	20	0.130 (±0.01)	0.046 (±0.026)	86.67	30.67
100	0	0.132 (±0.003)	0.028 (±0.006)	90.37	19.44

^a^Conversion = (Observed gas consumption/Expected gas consumption from CSMGem)*100.

^b^Experiments conducted at ~5300 kPa (@275 K) and all other experiments were perfomed at ~3300 kPa gas pressure.

**Figure 2:**
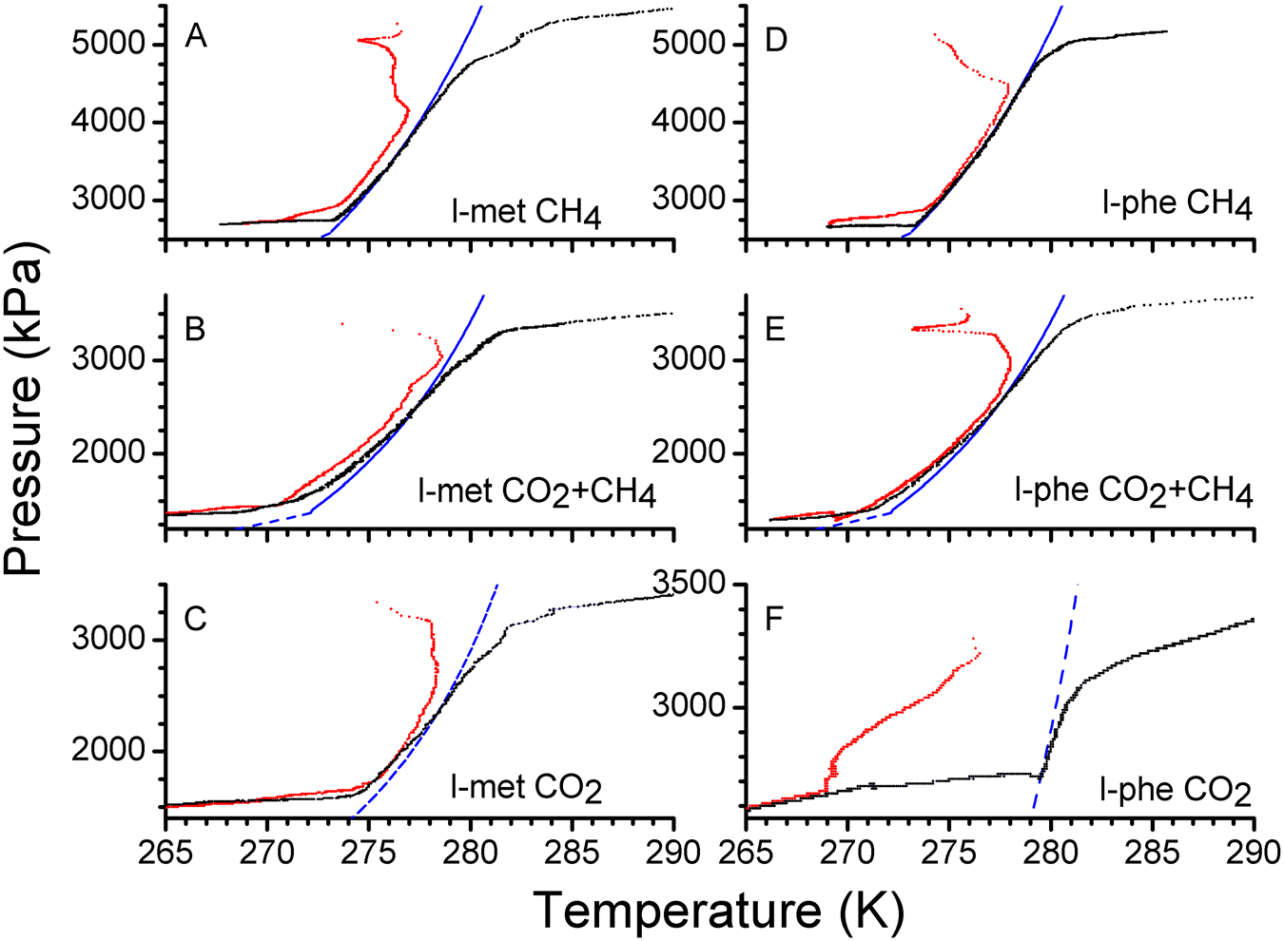
Representative pressure – temperature (p-T) trajectories for hydrate forming systems with 0.5 wt% l-methionine (l-met) (**A**–**C**) and l-phenylalanine (l-phe) (**D**–**F**). The red and black coloured dots are the recorded behaviour during the cooling and warming cycles. The blue coloured line represents the phase boundary curve computed from CSMGem model.

Figure 2 shows a representative pressure-temperature trajectory for each hydrate-forming system. The red and black dots indicate the recorded behaviour during cooling and thawing cycles; while the blue coloured line is the phase boundary curve computed using CSMGem^1^. Initially, the aqueous solution was cooled to 273–275 K, and then the reactor was pressurised with the guest molecules. Subsequently, the hydrate formation was evidently observed by the local temperature increase. Over a period the pressure was decreased significantly, and it follows the phase boundary curve. The hydrate-forming system was kept for about 10–12 hours in the temperature range 275–270 K to allow maximum hydrate conversion. After that, the temperature was decreased to 265 K. The hydrates were dissociated by increasing the temperature rapidly (6–8 K/h) and the gas consumed during hydrate growth process was fully recovered. In practice, the hydrate dissociation is measured with slower temperature ramp, i.e., 0.5 to 1.0 K/h, to determine phase stability points. Faster temperature ramping, however, leads to measurable deviations from computed phase boundary curve^22^. The primary goal of this study is to probe gas hydrate formation ability in the presence AAs, and not on the dissociation behavior. However, the temperature ramping was slower for the experiments conducted for their dissociation in meta-stable region.

### *Ex-situ* Characterisation of Gas Hydrate

All the hydrates were characterised using powder x-ray diffraction (PXRD) and Raman spectroscopic methods. After completion of hydrate growth, the reactor vessel was cooled to 150 K by placing it in liquid nitrogen, and the residual gas pressure was removed. A portion of the gas hydrate sample was collected for *ex-situ* spectroscopic analysis. Figure SI-1 (Supplementary Fig. 1) shows the XRD pattern of hydrate samples recorded at 150 K. A fine powder of the hydrate sample was prepared at liquid nitrogen environment. Blue and red coloured bars indicates the computed positions for the cubic hydrate and hexagonal ice respectively. In all the cases, the PXRD pattern could be indexed by the CheckCell^28^ programme with the space group *Pm3n* and the fitted unit cell parameters are in good agreement with earlier reports^29,30^. The PXRD pattern recorded for CH_4_ and CO_2_ hydrates, prepared in stirred configuration without amino acids, are shown in the Supplementary Information as SI-2a,b, respectively.

Simillarly, characteristic Raman features of CO_2_ and CH_4_ molecules encased in the hydrates synthesised in the presence of l-met and l-phe are shown in SI-3. Observed Raman modes around 1277, 1380 cm^−1^ and 2905, 2915 cm^−1^ respectively are due to CO_2_ and CH_4_ molecules occupying the cages of sI hydrates. The characteristic Raman spectral features of CO_2_ and CH_4_ molecules in hydrate phase, without amino acids, are shown in SI-4. The characteristic methane stretching mode at 2905 cm^−1^ is due to the guest encased in 5^12^6^2^ cage of sI, while the one at 2915 cm^−1^ is due to CH_4_ molecule trapped in 5^12^ cage^31,32^. However, the characteristic modes of encased CO_2_ molecules are due to fermi resonance and assigning them to different cages of sI is difficult task^33,34^. Absence of hot bands and broader fermi-doublet in the hydrate phase, in comparison with vapor phase, indicates the encasing of CO_2_ molecules in hydrate cages^33^.

### Gas Uptake kinetics in Hydrate

**Figure 3:**
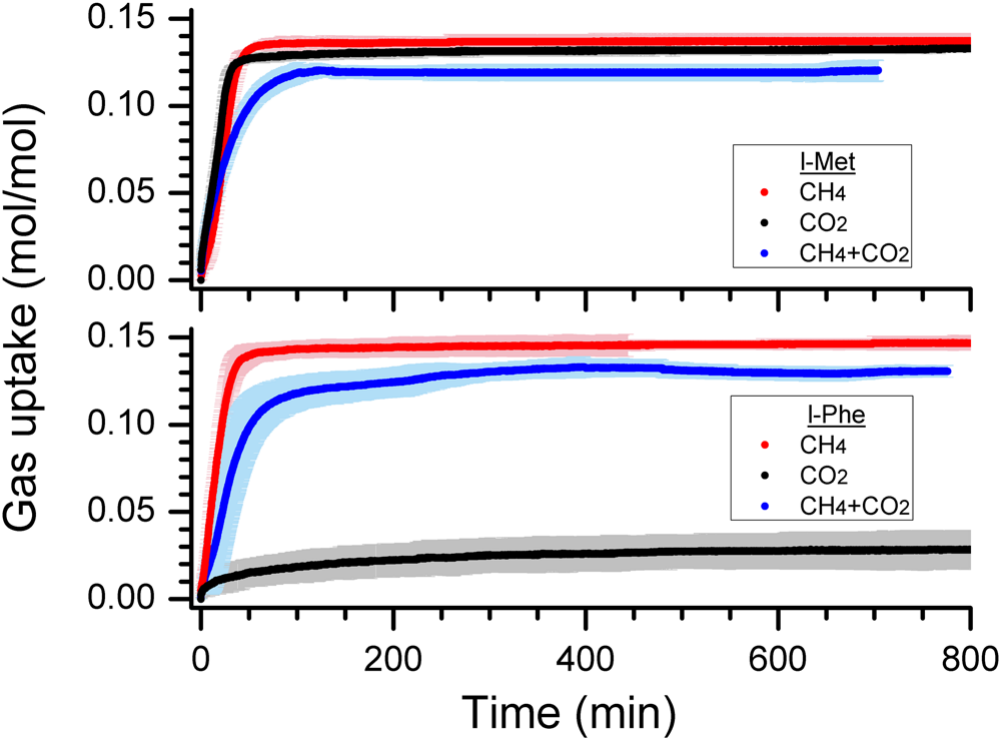
The gas uptake kinetics in the hydrates synthesised with 0.5 wt% l-methionine (l-met) (Top) and l-phenylalanine (l-phe) (Bottom). The red and black coloured dots represent the observed behaviour in CH_4_ – H_2_O and CO_2_ – H_2_O systems, while the blue coloured dot is for the gas mixture of CO_2_ + CH_4_ (54:46). The shaded portion indicates the standard deviation from repetitive gas uptake measurements.

Figure 3, shows the gas uptake kinetics during hydrate formation in l-met and l-phe systems in the time span of 800 min. As said, the hydrate nucleation point is seen by a decrease in the pressure and an increase in the local temperature. The zero time corresponds to hydrate onset point. The shaded portion of the gas uptake curves represents the standard deviation of repeated measurements. The ideal unit cell composition of sI gas hydrates is 6(5^12^6^2^)•2(5^12^)•46H_2_O. Thus the maximum gas consumption in ideal conditions is 0.174 mol/molH_2_O. Practically it is difficult to achieve 100% cage occupancy, and hence the gas hydrates become non-stoichiometric complexes. The cage occupancy of CH_4_ molecules is more than that of CO_2_, because of the molecular size. The CH_4_ can comfortably fit into both the cages, while CO_2_ is predominantly encased in the large cages^34^. Thus the calculated gas consumption for CO_2_ hydrates is less. In fact, the calculated gas consumption for these systems, with CO_2_, CH_4_ and gas mixture (54% CO_2_ + 46% CH_4_), is 0.146, 0.159 and 0.154 mol/molH_2_O. The gas uptake in the hydrate-forming systems with l-met is rapid and high, indicating the faster and efficient water to hydrate conversion. The average gas consumed in this system with CO_2_, CH_4_ and mixed gases are 0.132 (90.37%), 0.137 (86.28%) and 0.120 (77.96%) mol/molH_2_O. Similarly, the gas uptake in hydrate forming system with l-phe is following: 0.028 (19.44%), 0.147 (92.25%) and 0.131 (84.73%) mol/molH_2_O.

Another significant issue in gas storage applications in the form of hydrates is the growth kinetics. Faster kinetics is advantageous for increasing the process efficiency. The water to hydrate conversion in the absence of any promoters is negligible under non-stirred conditions. However, the addition of small amount (0.5 wt%) of the amino acids to the water not only helped in promoting hydrate conversion but also the gas uptake is very much rapid. As shown in Fig. 3, the gas consumption in hydrates is rapid in methane hydrate systems with both l-met and l-phe. In fact, it took 40 and 36 min respectively to achieve 90% of gas consumption. Similarly, the time required for 90% of CO_2_ consumption in the l-met system is 31 min. The gas consumption rate in all these systems is about 4.5 m.mol/molH_2_O/min. Thus, these two amino acids are useful for efficient and rapid gas storage of CH_4_ and CO_2_ gases in the form of hydrates.

Surfactants like, sodium dodecyl sulfate (SDS), sodium dodecyl benzene sulfonate (SDBS), are widely used to accelerate the hydrate formation^35–37^. In general, surfactants help in improving the hydrate formation kinetics and the induction time is significantly less compare to pure hydrates, even when they are present in smaller quantities (i.e., 0.05 wt%). We synthesized CH_4_ and CO_2_ hydrates using 0.5 wt% SDS, using the similar experimental procedure. As shown in Figs SI-5 and SI-6 CH_4_ and CO_2_ hydrates were formed under the non-stirred configuration in the presence of SDS, and the hydrate growth kinetics is also similar to l-met system. A measurable delay is undoubtedly noticed in the hydrate nucleation in SDS systems, while it is instantaneous in the l-met system. Substantial foaming in SDS containing systems makes it difficult to reuse the hydrate-forming solutions, while such effect is not present in AAs (see Fig. SI-7).

Some specific properties like THI and higher gas uptake in hydrate phase, with a rapid rate, using amino acids are difficult to understand at the molecular level. The zwitterionic nature of amino acids triggers interaction with hydrogen-bonded water molecular network. Sa *et al*.^16^, observed that some amino acids like l-val, l-ala and gly incorporated in the hydrate lattice and expand the unit cell. However, the extent of lattice expansion saturated at different wt%, e.g., gly 9.12 wt% (2.2 mol%) and l-ala 2.47 wt% (0.5 mol%). Therefore, presence of amino acids above a certain limit tend to crystallise among themselves, and weakly interact with hydrate water. Additionally, the hydrophobic index; nature and length of side chain also play a critical role. Further, visually we observed that the solution creeps all around the walls and to the top part of the vessel. Similar hydrate growth behaviour is described using some surfactants in the literature^37^. This effect ensures enhanced interaction between water molecules, via capillary action, and gas molecules, thus promoting a faster and efficient hydrate conversion in the presence of AAs.

### Gas Hydrates for transportation application

**Figure 4:**
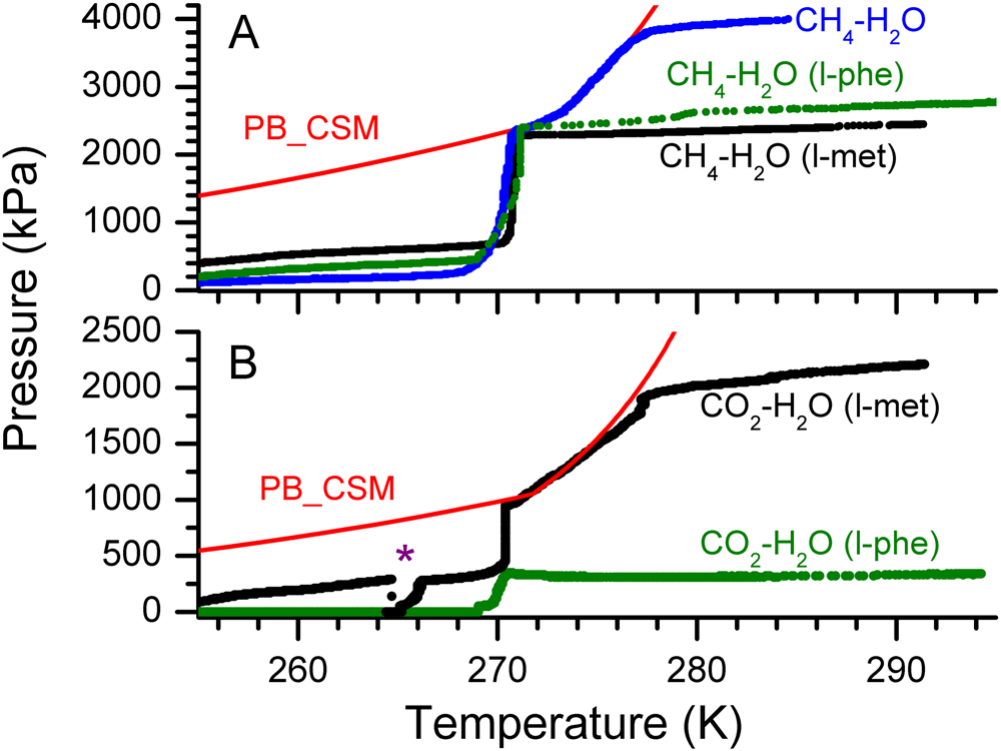
The pressure – temperature (p-T) trajectories for the hydrates with 0.5 wt% l-methionine (l-met) and l-phenylalanine (l-phe) in the thermodynamically meta-stable region. The top and bottom graphs show the behaviour in CH_4_ – H_2_O and CO_2_ – H_2_O systems respectively. The computed phase boundary curve for each system is shown by the red line. The black and green coloured dots represent the collective pressure built-up from the hydrate metastable region. The blue coloured dots on the top graphs show observed p-T trajectory in CH_4_ – H_2_O system without any amino acids, which helps as a control experiment.

Gas hydrates, particularly, with methane or carbon dioxide are stable out of their thermodynamically stable regions. Although the reason for such peculiar property is still not understood, the hydrates are stable for more prolonged periods even at atmospheric pressures by preserving them sub-zero temperatures. This property is popularly known as “self- or anomalous-“ preservation effect^7,8^. Preservation of carbon dioxide hydrates in the presence of sugar, under freezer conditions, has useful applications in food and beverage industry^38^. In Fig. 4, we plot the dissociation behaviour of CH_4_ – H_2_O and CO_2_ – H_2_O hydrate systems in the presence of l-met and l-phe, along with computed phase boundary curves using CSMGem^1^. The reactor vessel was equilibrated to the atmospheric pressure by removing the residual methane gas at a lower temperature, and the hydrate system firmly is in its metastable state. As discussed rapid dissociation (faster heating rate) may cause a measurable deviations from the thermodnamic phase stability curve. Therefore, we conducted these experiments with slower heating rates (~1.0 K/h). The pressure build-up is remarkably sluggish in CH_4_ – H_2_O system when the temperature is below 268 K. The gas increases rapidly in the temperature window of 268 to 271 K. Pressure inside the reactor reached to 2350 kPa at 270.8 K, which is evidently a phase boundary point for pure methane hydrates. After that, the gas release by hydrate dissociation is primarily driven by the thermodynamic conditions. For more clarity, the gas release behaviour is compared with pure methane hydrate system, along with phase boundary curve computed using CSMGem^1^. Similar behaviour is observed in CO_2_ – H_2_O system also. As already shown, the hydrate conversion efficiency of CO_2_ – H_2_O system is high in the presence of l-met; while it is considerably lesser in association with l-phe (see Fig. 4B). Therefore the gas released due to hydrate dissociation is less in l-phe system. The dissociation tendency of CO_2_ – H_2_O system is similar to the CH_4_ – H_2_O system in l-met. Again the gas release is rapid in small temperature window. When the collective pressure due to hydrate dissociation equals to the phase boundary point, further hydrate dissociation is fundamentally controlled by the thermodynamic perturbations. The star mark indicates the instant where the pressure vessel was degassed again. However, the hydrates dissociate rapid, and the pressure was increased to about 270 kPa at 266.3 K. Nevertheless, the critical pressure increase is in the small temperature window of 268–271 K. Although the exact mechanism for self-preservation effect remained uncertain, and most acceptable explanation is that “the formation of temperature dependent solid-like layer at the hydrate interface, and this will be an additional heat & mass transfer barrier for the diffusion of gas molecules from the hydrates^39^”. Therefore, specific hydrates show prolonged stability in self-preservation window. Present result demonstrates the ability of methane and carbon dioxide hydrates synthesised along with amino acids, which help in significantly faster and efficient hydrate conversion, to remain as a stable phase in its meta-stable region.

### Gas Hydrates with CO_2_, CH_4_ and gas mixtures

Recently the amino acids are being used to promote the hydrate formation rate, particularly under low concentrations^22–24^. The exact nature for its increased gas uptake ratio is still obscure. Nevertheless, experimental results from different groups vindicated the effect of thermal inhibition for some amino acid solutions is significantly less, while the hydrate formation rate, even under non-stirred configuration, is higher at lower concentrations. Typically, the water to hydrate conversion is significantly less and ineffective in the absence of additives. Aforementioned is further less under non-stirred geometry. As shown in Fig. 2, the observed pressure drop in H_2_O – CH_4_ system with two amino acids is around 2600 kPa. While in H_2_O – CO_2_ system it is nearly 1750 and 800 kPa in hydrate forming systems with l-met and l-phe, respectively. Observed p-T trajectory (Fig. 2) during cooling and thawing cycle, along with CSMGem computed phase boundary curve indicates that the pressure drop is indeed predominantly due to the phase change to hydrates. Further, characteristic PXRD pattern and Raman spectroscopic as shown in Figs SI-1 and SI-3 confirm the dominance of hydrate phase, excepting the H_2_O – CO_2_ system with l-phe. In this system hydrate phase is only about 19.44% and hexagonal ice contribution is dominant in PXRD (Fig. SI-1). We estimated the cage occupancy values for CO_2_ – H_2_O system using similar feed compositions, i.e., 0.345 mol CO_2_ and 1.6 mol H_2_O, using CSMGem and found them as 0.5399 (for 5^12^) and 0.9657 (for 5^12^6^2^) at 275 K. The average gas uptake expected from hydrates with such cage occupancies is about 0.146 (±0.002) mol/molH_2_O. Similarly, for CH_4_ – H_2_O system the cage occupancy values are 0.8363 (for 5^12^) and 0.9469 (for 5^12^6^2^) at 275 K (0.510 moles of CH_4_ and 1.6 moles H_2_O). The experiments in CH_4_ – H_2_O are at higher pressure, as the equilibrium pressure for this system is always greater than CO_2_ – H_2_O system (see Fig. 1). The average methane gas consumption expected based on CSMGem model is 0.159 (±0.002) mol/molH_2_O. Observed gas uptake in CH_4_ – H_2_O and CO_2_ – H_2_O systems with l-met (0.5 wt%) is 0.137 and 0.132 mol/molH_2_O; corresponding to 86.3% and 90.4% hydrate conversion. Liu *et al*. reported 0.157 mol/molH_2_O (or 140 mg/g) methane gas consumption in l-met (0.5 wt%) system at somewhat higher gas pressure (9.0 MPa)^24^. Further, Cai *et al*. reported 0.141 mol/molH_2_O (or 346 mg/g) CO_2_ gas consumption in l-met (0.5 wt%) system^22^. The gas uptake values obtained in the present study are comparable to these results. Similarly, the CH_4_ and CO_2_ gas consumption in the hydrate-forming system with l-phe is 0.147 and 0.028 mol/molH_2_O respectively. Liu *et al*. reported that 0.5 wt% AA systems, such as l-met, l-phe and l-tryptophan are good hydrate promoters for CH_4_ – H_2_O system, with rapid and higher gas consumption^24^. In contrast, the methane gas uptake in AA systems with l-serline, l-aspartic acid and l-proline is negligibly small in CH_4_ – H_2_O system^24^. Sa *et al*., have also reported l-alanine as an active inhibitor for CO_2_ hydrate system, whereas it has no influence on CH_4_ hydrates and they attribute the plausible reasons to nature and length of side chain; the hydrophobicity index and abnormal interactions with water molecules^14,17^. The present study also shows that the l-phe is a suitable promoter for CH_4_ hydrates, but it has a marginal effect on CO_2_ hydrates. On the other hand, Bavoh *et al*.^21^, have noticed that some amino acids such as arginine and valine are THIs for methane hydrates. However, the total gas uptake during the hydrate formation is higher, respectively, by about 2.5 and 10 times higher than pure water system. The authors claim that “the higher methane hydrate promotion of valine than arginine is due to the inclusion of methyl-R groups in valine into the hydrate cages”^21^. Further, the presence of some amino acids such as l-val, l-cys and l-met promotes gas uptake in CO_2_ – H_2_O system, whereas, the gas uptake is significantly less for l-thr and l-phe containing systems^40^. In other words, some of the amino acids are suitable promoters for hydrate-forming systems, and apparently, there is no simple correlation between the properties mentioned above of amino acids such as hydrophobicity index, nature and length of the side chain, and the hydrate promotion effect. It is difficult to assign a particular reason for such contrasting behaviour for an amino acid towards CH_4_ and CO_2_ systems. One salient point, the solubility of l-met and l-phe is higher (~50 g/L) in acidic and basic solutions, respectively, whereas, the solubility in water is reduced to 33.8 and 29.6 g/L. But both of them have hydrophobic side chains and the iso-electric points are also close, i.e., 5.74 and 5.48, respectively. The concentration of aqueous AA solutions in these experiments is at least one order of magnitude lesser. We observed that the presence of l-phe has a lesser influence on hydrate formation, particularly the gas uptake during hydrate formation process is similar to CO_2_ – H_2_O system^40^. Further, despite the larger guest-size of CO_2_ as compared to CH_4_ molecules, the lattice constant for CO_2_ hydrates is smaller by about 0.092%^29^. Similar lattice shrinkage by 0.084% is reported by Everett *et al*.^30^. Thus the CO_2_ molecules have stronger guest-host interactions. Noticeably, the total gas consumption, estimated by CSMGem, is lesser for CO_2_ hydrates as compared to CH_4_ hydrates. The shrinkage in unit cell plausibly contributes to lesser cage occupancy. We observe the lattice shrinkage of 0.11% for l-phe, while l-met system has opposite effect. Thus the affinity for l-phe system is more similar to pure water system. The concentration of CH_4_ and CO_2_ hydrate phases in the l-phe system, with 0.147 and 0.028 mol/molH_2_O is about 92.3% and 19.5% respectively. Nevertheless, such a noticeable discrepancy in water to hydrate conversion efficiency could be utilised for separating CH_4_/CO_2_ gas mixtures.

We also conducted experiments with CO_2_ and CH_4_ gas mixtures (54% CO_2_ + 46% CH_4_) and kept experimental pressure around 3300 kPa, as schematically shown in Fig. 1. Observed pressure drop is nearly similar (~2100 kPa) in both l-met and l-phe systems. By following the CSMGem model, the cage occupancy values for CO_2_ molecules can be estimated as 0.2354 (5^12^) and 0.7263 (5^12^6^2^). Similarly, for CH_4_ molecules, they are 0.4824 (5^12^) and 0.2315 (5^12^6^2^). Understandably the CO_2_ molecules prefer to occupy the large (5^12^6^2^) cages. In such gas mixtures, the smaller cages (5^12^) are filled by the CH_4_ molecules. It is worth noticing that PXRD pattern obtained with both amino acids (Fig. SI-1) shows dominant sI structure features and the behaviour is similar to CH_4_ – H_2_O system. The Raman spectroscopic signatures also matched with encased CO_2_ and CH_4_ molecules (Fig. SI-3). It is hard to distinguish Raman signatures for CO_2_ molecules encased in 5^12^ and 5^12^6^2^ cages of sI structure^33,34^. However, this is much simpler in case of CH_4_ molecules. The characteristic Raman bands for 5^12^ and 5^12^6^2^ cages are found around 2915 and 2905 cm^−1^. The Raman spectral features also help in estimating the cage occupancy and hydration number values based on intensity ratios of the characteristic features. The intensity of Raman band due to the large cage is ideally three times more intense than that due to the small cage, because of the unit cell of methane hydrate consists of six large cages and two small cages. However, in practice, this is more than three since the hydrates are non-stoichiometric complexes with cage occupancy values are always less than one. Indeed in case of CH_4_ – H_2_O systems, we obtained the area ratio of Raman bands in l-met and l-phe systems as 3.35 and 4.35 respectively. On the other hand, the ratio is 1.30 and 1.69 for mixed gas systems with l-met and l-phe. Interestingly, the cage occupancy values computed from CSMGem model results in this ratio as (1.33 ± 0.095) and experimental observations are closely comparable. Again by following the cage occupancy values estimated from CSMGem model one can expect gas consumption of 0.154 (±0.005) mol/molH_2_O in the mixed gas system. Experimentally observed values are 0.120 and 0.131 mol/molH_2_O in l-met and l-phe systems respectively, which corresponds 78% and 84.7% hydrate conversion. Although, such higher gas consumption in the l-met system is understandable because it can help in efficient storage of both CO_2_ and CH_4_ gases in the form of hydrates. However, the gas consumption in l-phe (0.131 mol/molH_2_O) is significantly large. The gas consumption in CO_2_ –H_2_O and CH_4_ – H_2_O system with l-phe is 0.028 and 0.147 mol/molH_2_O respectively. Unlike l-met, l-phe do not help in promoting CO_2_ gas storage in the form of hydrates. The linear extrapolation considering feed gas composition (i.e., 0.028*0.54 + 0.147*0.46) also results in a significantly lesser value 0.084 mol/molH_2_O.

**Figure 5:**
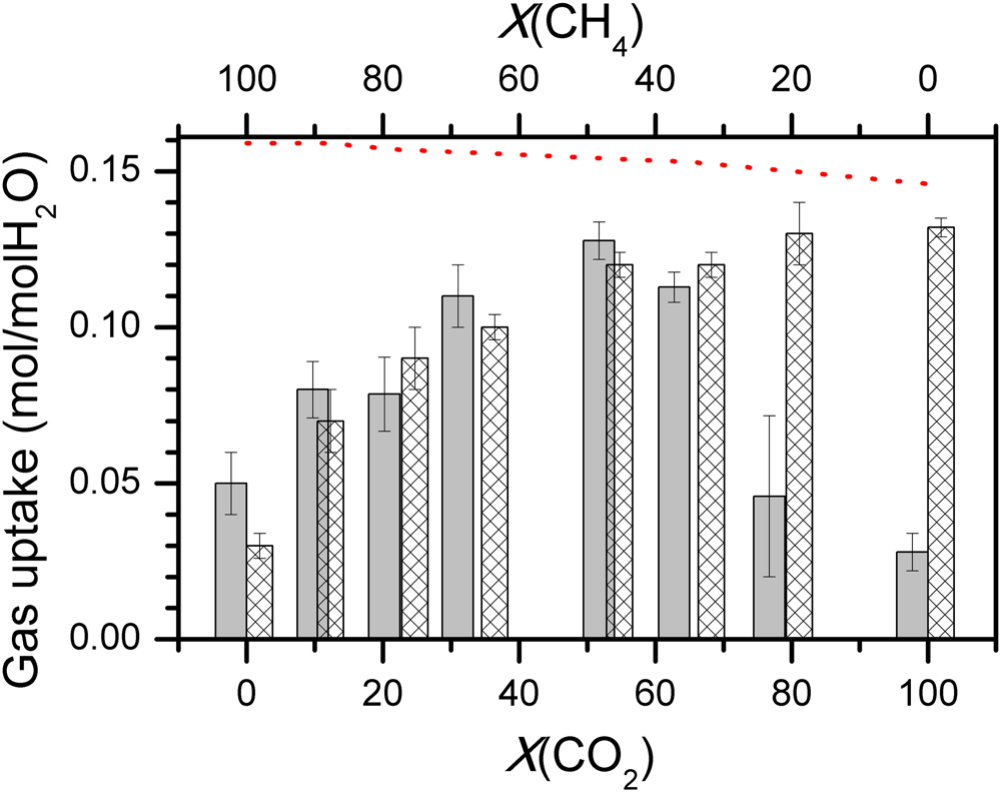
A graph showing measured gas uptake due to the hydrate formation with 0.5 wt% l-methionine (l-met) (bars with Sparse pattern) and l-phenylalanine (l-phe) (filled bars) using CO_2_ and CH_4_ gas mixtures. All the experiments were conducted with initial gas pressure ~3300 kPa. The top and bottom scale represent percent mole fraction of CH_4_ and CO_2_ in the feed gas. Maximum gas uptake expected in the hydrate-forming system with gas mixtures, using CSMGem model, is shown as dotted line in red.

We conducted some more experiments with the gas mixtures for understanding the role of l-met and l-phe systems in the hydrate conversion process. As stated the experiments on CH_4_ – H_2_O were with higher gas pressures to maintain the driving force (i.e., the difference between the experimental pressure and the equilibrium pressure) comparable to CO_2_ – H_2_O system. Therefore, the initial total pressure for hydrates with gas mixtures was maintained at the same level as CO_2_ – H_2_O system. In other words, the molar ratio of guest and host molecules were preserved at 0.19 (±0.02) in all the runs. The driving force for CH_4_ – H_2_O system under these conditions is 482 kPa, and this progressively increases to 1900 kPa for mixed gas systems. The gas uptake in hydrates by systematically varying the molar concentration of CO_2_ in gas mixtures is shown in Fig. 5. The aqueous solution here contains 0.5 wt% of l-met (histograms with sparse pattern in Fig. 5) and l-phe (filled histograms in Fig. 5). Observed methane gas consumption of 0.03 and 0.05 mol/mol H_2_O is significantly larger, particularly at smaller driving force (482 kPa). Methane hydrates will not form in non-stirred configuration with such low driving force. In that sense, these amino acids are helping in methane hydrate formation. Also, the dotted line in this figure shows the maximum gas consumption expected from the cage occupancy values computed from CSMGem model^1^.

The gas uptake in l-met system is progressively increased by increasing the CO_2_ content in the feed gas and eventually reaches a plateau indicating the saturation in hydrate conversion. This kind of behaviour has been reported in literature for multi-component gas hydrate systems^41,42^. On the other hand, variation in the gas uptake with l-phe is interesting. As shown in Fig. 5, the total gas uptake in these two systems is nearly identical in the methane-rich feed gas. Understandably, these two amino acids help in efficient storage of methane gas in the form of hydrates. However, the same behaviour is continued until CO_2_ content in feed-gas reaches to a level of ~65%. At this point, the total gas consumption with both the amino acid systems accounts for 71% (l-phe) and 78% (l-met) hydrate conversion. However, the situation dramatically changed in feed gas rich in CO_2_ (~80%). The gas consumption in such case is equivalent to about 30% (l-phe) and 87% (l-met) hydrate conversions. As observed, l-met systems assist as an efficient promoter for hydrates with both CO_2_ and CH_4_ guest molecules, and thus the gas uptake in the mixed feed gas is by and large on expected trends. However, l-phe is a weak promoter for CO_2_ hydrates and hence lesser gas uptake in the CO_2_ rich feed gas is not surprising. However, the higher gas uptake (comparable to l-met) in other feed gas compositions is remarkable, and evidently, it is not following the linearity of the constituent guest composition. The l-phe system is helpful in encasing the CH_4_ molecules.

## Conclusions

In summary, we systematically examined hydrate formation behaviour of the aqueous solution with low (0.5 wt%) weight fraction of two amino acids namely, l-methionine and l-phenylalanine, using CH_4_ and CO_2_ molecules as guests. Experiments were conducted in isochoric and non-stirred conditions which could be easily adaptable for large-scale applications. The l-met system promotes faster and efficient hydrates with both the guests, while the l-phe systems is a suitable promoter for methane hydrates only. The hydrates synthesised in the presence of these amino acids are remarkably stable in the meta-stable window. Thus they are very beneficial in storage and transportation applications. The contrary affinity of l-phe system for CO_2_ and CH_4_ hydrates make it attractive for the separation of CH_4_ gas from CO_2_+CH_4_ gas mixtures.

## Experimental Section

The amino acid powders were purchased from M/S Sigma Aldrich and were used as received. De-ionized ultra-pure water (Millipore – type 1) was used, and dissolved gases were removed by evacuation. High purity (99.95%) methane and carbon dioxide were procured from M/S Bhuruka Gas Company.

### Apparatus

The central part of the experimental set up is an SS-316 cylindrical vessel, which can withstand gas pressures up to 20 MPa, and volume of the vessel was 250 mL. Cold fluid (water + glycol mixture) was circulated the vessel with the help of a circulator to bring and maintain the temperature inside the cell at the desired level. A platinum resistance thermometer (Pt100) was inserted into the vessel to measure the temperature with an accuracy of ±0.2 K. The pressure in the vessel was measured with the pressure transducer (WIKA, type A-10 for pressure range 0–25 MPa with ±0.5% accuracy).

### Procedure

The reactor vessel was filled with 29 g of the aqueous solution consisting of 0.5 wt% amino acid. The reactor vessel was cooled to the desired experimental temperature and was kept for equilibration. The vessel was charged with desired gas to the experimental pressures using Teledyne ISCO syringe pump. Then, the reactor was isolated from the ISCO pump/gas tank by closing the gas inlet valve. Subsequently, the hydrate formation was detected by a sharp pressure drop. The insignificant head-pressure drop in the reactor over a longer duration indicates the saturation in hydrate conversion. The temperature and pressure were logged for every 30 seconds of the time interval. The molar concentration of methane gas (Δ*n*H, *t*) in the hydrate phase during the experiment at time *t*, is defined by the following equation:

1 $${\rm{\Delta }}n{\rm{H}},t=ng,0\,-ng,t=({P}_{0}V/{Z}_{0}{{\rm{RT}}}_{0})-({P}_{t}V/{Z}_{t}{{\rm{RT}}}_{t})$$

where, *Z* is the compressibility factor calculated by the Peng-Robinson equation of state. The gas volume (*V*) was assumed as constant during the experiments, i.e., the volume changes due to phase transitions were neglected. *n*g,0 and *n*g,*t* represent the number of moles of feed (methane) gas at zero time and in the gas phase at time *t*, respectively. P and T are measured pressure (vapor phase) and temperature of hydrate forming solution.

### Raman Measurements

The Micro-Raman (Horiba, T-64000) measurements were carried out using 514.5 nm from an air-cooled argon ion laser as excitation source. The Raman spectrum at low temperature was collected by mounting the sample onto a LINKAM FTIR 600 stage. The stage was pre-cooled to about 153 K, and a small amount of hydrate under atmospheric pressure condition was used for further study. Only during the data accumulation, the laser was focused onto the sample using a 50 × lens. The observed profiles were fitted into several Lorentzian components using GRAMS/3 software, and best-fitted parameters were used in data interpretations. Peak position, width and intensity were varied as free parameters and were allowed to vary to optimise the peak fitting routine. From the fitted curves, the ratio of the integrated intensities of large to small cages of the Raman spectrum was calculated.

### Powder x-ray diffraction measurements

The powder XRD was measured at 150 K on a Bruker (Advance D8) diffractometer with Cu- radiation (wavelength 1.5406 Å) in the θ/2θ scan mode. A cryo-temperature stage Anton-Paar (TTK-450) is fitted on the diffractometer. The experiments were carried out in step scan mode with a dwell time of 0.5 s and step size of 0.02°. The PXRD pattern was collected in the range 2θ = 8 to 60°. The PXRD pattern indexing and cell refinement were obtained using the Checkcell programme.

## Electronic supplementary material

Supplementary Information
