# Supplementary material for: Clathrate Hydrates of Greenhouse Gases in the Presence of Natural Amino Acids: Storage, Transportation and Separation Applications
Source: Sci Rep. 2018 Jun 4;8:8560. doi: 10.1038/s41598-018-26916-1 (PMC5986743; doi:10.1038/s41598-018-26916-1)
Supplement: Supplementary file 1 — Supplementary Information [file 41598_2018_26916_MOESM1_ESM.pdf]

<u>Supplementary Information</u>

Clathrate Hydrates of Greenhouse Gases in the Presence of Natural Amino Acids: Storage, Transportation and Separation Applications

PINNELLI. S.R. Prasad,* and BURLA. Sai Kiran

Gas Hydrate Division, CSIR–National Geophysical Research Institute (CSIR–NGRI),

HYDERABAD – 500 007 (India)

Corresponding author's e-mail: psrprasad@ngri.res.in; Phone: +91 40 2701 2710;

Fax: +91 40 2717 1564

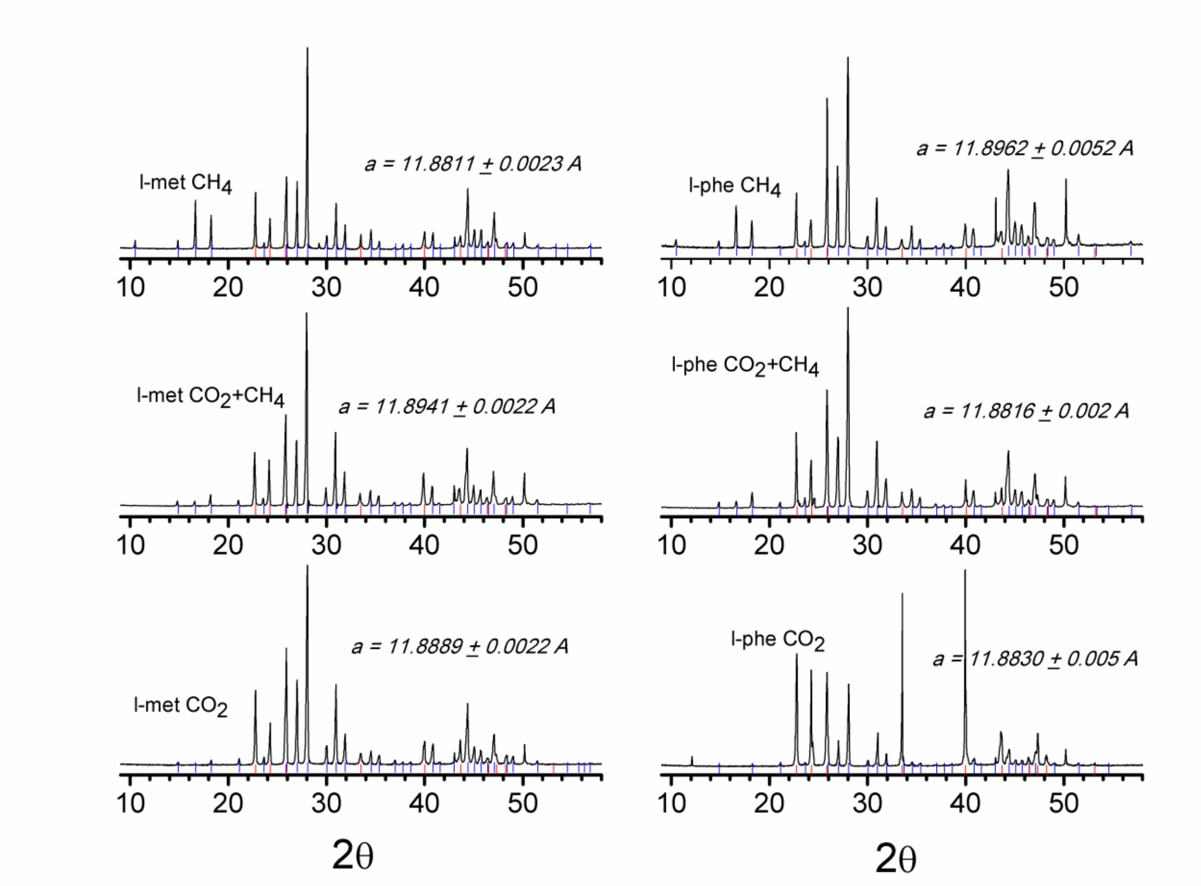

<u>Figure SI-1</u>: Powder x-ray diffraction pattern for the hydrates synthesised with 0.5 wt% l-methionine (l-met) (left-side) and l-phenylalanine (l-phe) (right-side). The red and blue coloured bars represent the computed positions for hexagonal ice and cubic hydrate phases. The Check-cell programme is used for the structural refinement. All the spectra were recorded at 100 kPa and 150 K.

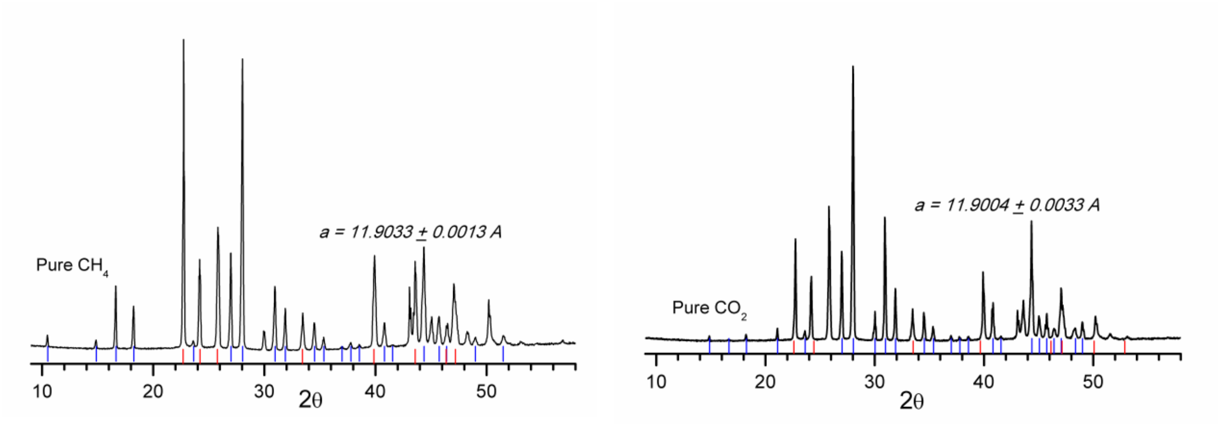

Figure SI-2: The powder XRD spectrum of pure CH_4 and CO_2 hydrates (without l-met or l-phe). Blue and red bars are the calculated peak positions for the hydrate (*Pm3n*) and ice (*P*6_3/*mmc*) phases respectively.

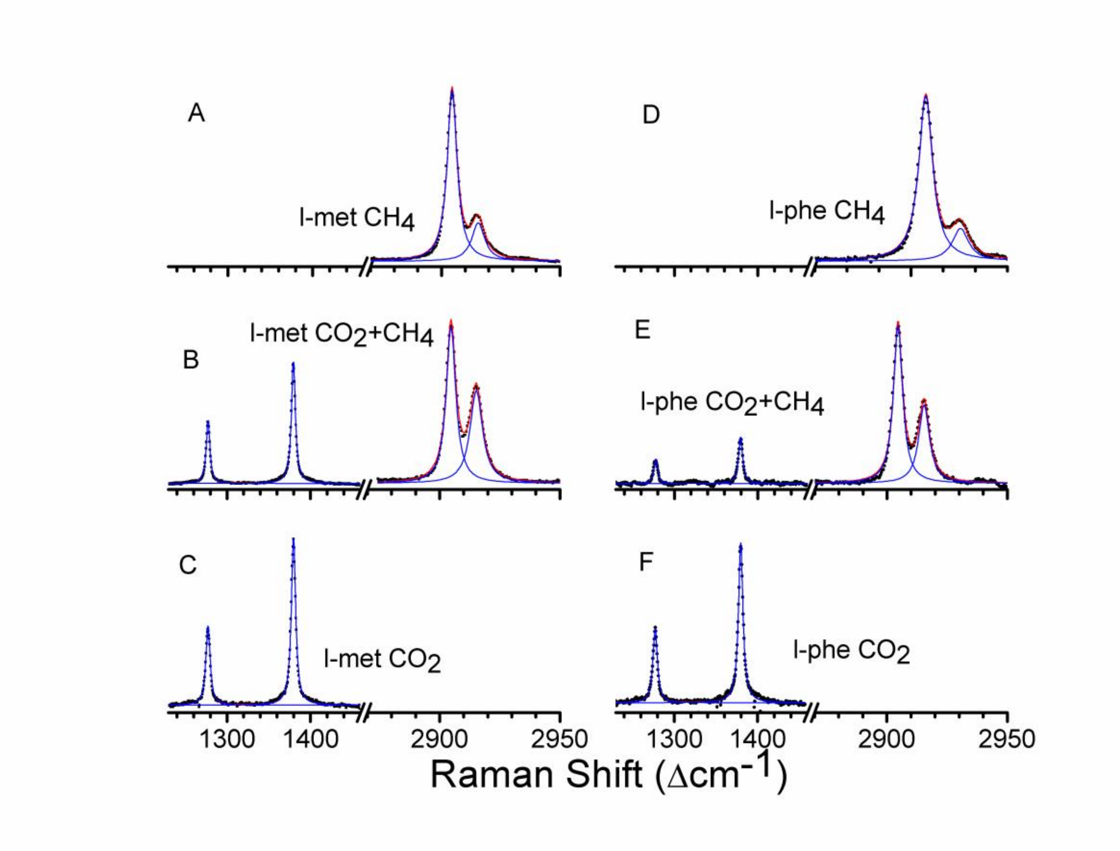

Figure SI-3: Characteristic Raman bands for the CO_2 and CH_4 molecules encased in the hydrates synthesised with 0.5 wt% l-methionine (l-met) (A,B,C) and l-phenylalanine (l-phe) (D,E,F). The thin blue coloured lines are the individual Lorentzian profiles, while the red coloured line is the combined profile for the experimental points. All the spectra were recorded at 100 kPa and 150 K.

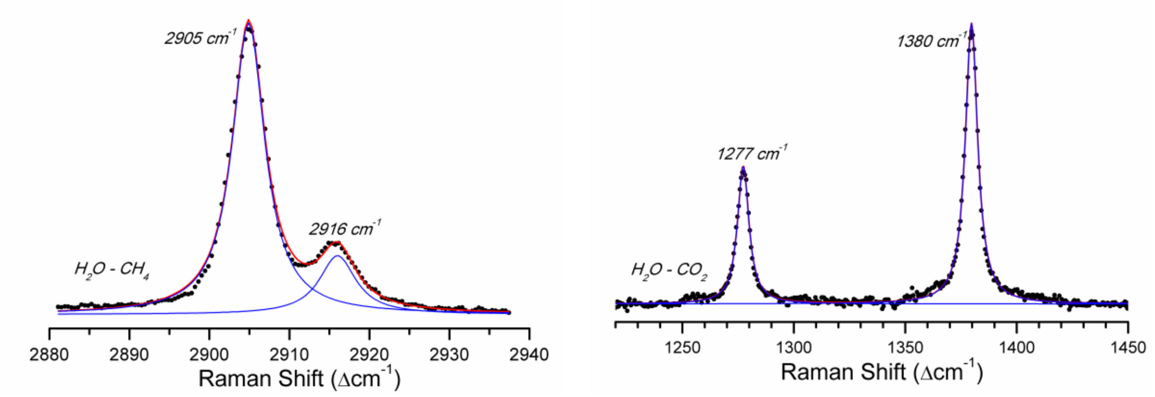

Figure SI-4: The characteristic Raman peaks for CH_4 and CO_2 molecules encased in sI hydrate (without l-met or l-phe) cages.

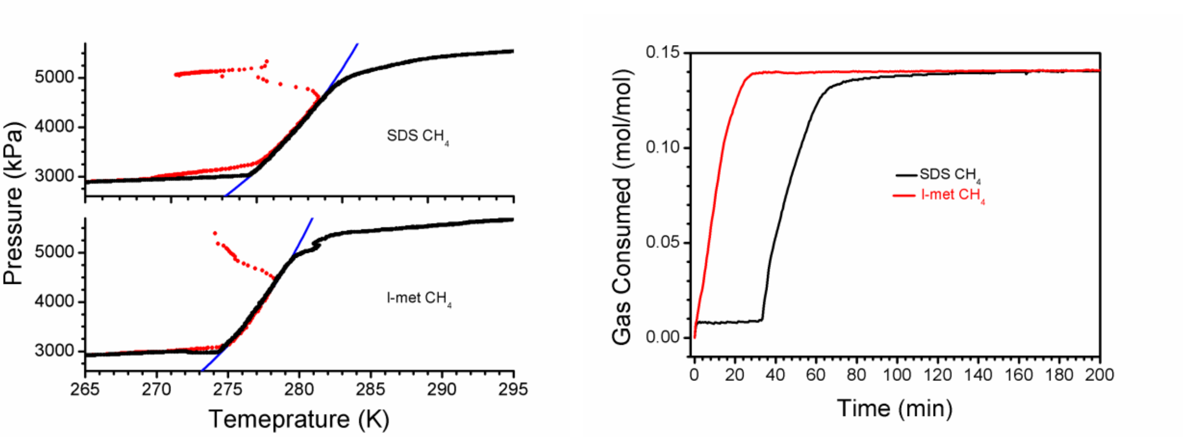

Figure SI-5: The pressure-temperature trajectories in cooling and thawing cycles using 0.5 wt% SDS and l-met in H_2O – CH_4 systems. The formation kinetics is shown in the figure on the right side.

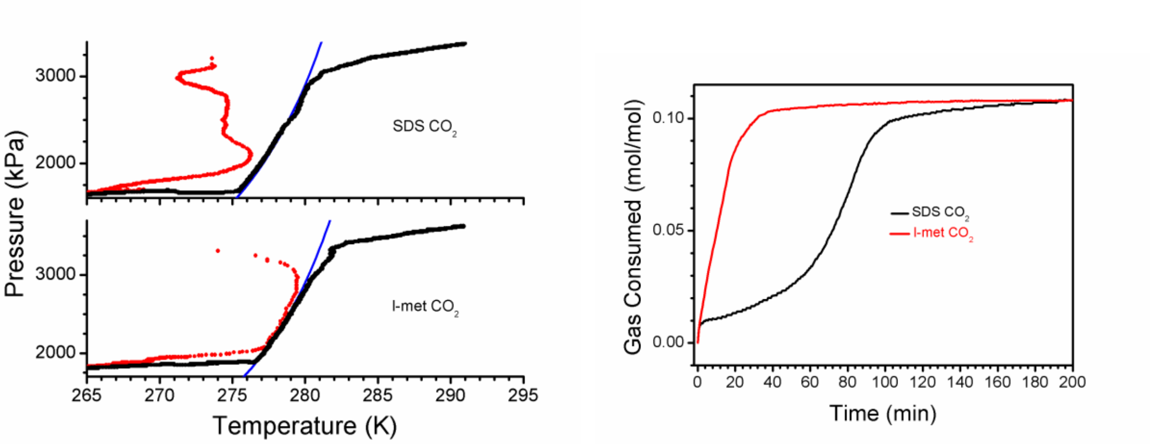

Figure SI-6: The pressure-temperature trajectories showing hydrate formation and dissociation in 0.5 wt% SDS and l-met in H_2O – CO_2 systems. The formation kinetics is shown in the figure on the right side.

Appearance of sample solution after dissociation of Gas Hydrate.

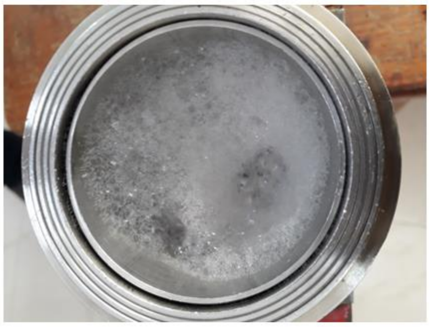

0.5 wt% SDS solution with CH_4 gas

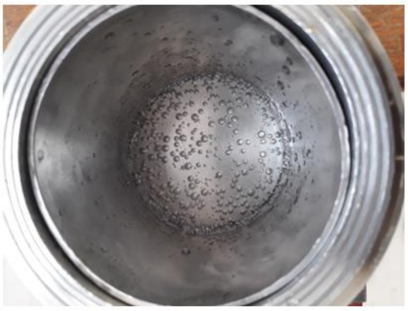

0.5 wt% l-met solution with CH_4 gas

Figure SI-7: Photographs showing the status of the solutions after hydrate dissociation. The foaming is predominantly observed only in 0.5 wt% SDS systems.
